# Evaluation of the Pint of Science festival in Thailand

**DOI:** 10.1371/journal.pone.0219983

**Published:** 2019-07-18

**Authors:** Bipin Adhikari, Phyu Hnin Hlaing, Matthew T. Robinson, Andrea Ruecker, Noel Hidalgo Tan, Nattapat Jatupornpimol, Rita Chanviriyavuth, Phaik Yeong Cheah

**Affiliations:** 1 Mahidol-Oxford Tropical Medicine Research Unit, Faculty of Tropical Medicine, Mahidol University, Bangkok, Thailand; 2 Centre for Tropical Medicine and Global Health, Nuffield Department of Medicine, University of Oxford, Oxford, United Kingdom; 3 Lao-Oxford-Mahosot Hospital-Wellcome Trust Research Unit (LOMWRU), Microbiology Laboratory, Mahosot Hospital, Vientiane, Laos; 4 Southeast Asian Regional Centre for Archaeology and Fine Arts (SEAMEO SPAFA), Bangkok, Thailand; 5 The Ethox Centre, Nuffield Department of Population Health, University of Oxford, Oxford, United Kingdom; IUMPA - Universitat Politecnica de Valencia, SPAIN

## Abstract

**Background:**

The Pint of Science festival is the biggest annual international science festival. In May 2017, we coordinated the first Pint of Science festival in Thailand and reported our initial reflections. Building on this work, we set out to evaluate more systematically events conducted in 2018.

**Methods:**

In 2018, we conducted Pint of Science events at four different locations in Bangkok. Overall, there were 18 talks held over six event-days in 2018. We administered 180 self-reported questionnaires as well as conducted 11 semi-structured interviews and a focus group discussion with audience members and speakers.

**Results:**

Of the 180 questionnaires handed out, 125 attendees completed the questionnaire. The majority of attendees came because they were interested in science (68.0%), to learn something new (46.4%) and to enjoy themselves (44.8%). Our qualitative results confirm the quantitative findings. In addition, speakers viewed that they benefited by improving their communication skills and having the opportunity to network with scientists and non-scientists. Speakers also mentioned that such events were a good means to engage with the public, can improve the visibility of their work and potentially attract more funding. To improve the Pint of Science activities, audience members suggested to include a more diverse range of topics, more collaborations with other local research institutions and to hold the event at larger venues.

**Conclusions:**

We conclude that Pint of Science was well received in Bangkok with recommendations to improve minor issues related to practicalities and logistics.

## Introduction

The Pint of Science festival is the biggest annual international science festival held over three days in May each year [1, 2]. The festival began in 2013 and was first held in fifteen pubs across three cities in the United Kingdom (London, Oxford, and Cambridge) [1, 3]. In May 2017, the Mahidol-Oxford Tropical Medicine Research Unit (MORU) coordinated the first Pint of Science festival in Asia [4]. The event which was held in Bangkok, Thailand, followed the standard Pint of Science festival program, with three event nights, each consisting of three different talks from researchers working locally [4].

Typically, Pint of Science events are held in a relaxed and enjoyable environment where members of the general public could meet and interact directly with scientists [1, 3]. Researchers across all fields of science and from all academic levels (PhD students, postdoctoral researchers and senior professors) give short, interesting, and interactive talks about their current research, while audience members get the opportunity to ask questions and have discussions with scientists and each other. The ethos of Pint of Science is similar to that of science cafés i.e. “a place where for a price of a cup of coffee or a glass of wine, anyone can meet to discuss the latest ideas of science that are impacting society” [5]. In recent years, science cafés and events of similar nature such as “Nerd Nite” have increased in number around the globe including in Southeast Asia such as in Thailand, Malaysia, Cambodia, Laos and Hong Kong [6–8].

Pint of Science Thailand (PoST) is one of a range of public engagement activities coordinated by the MORU, a Wellcome-funded research unit based in Bangkok. MORU’s portfolio includes targeted engagement around specific research studies [9–17], activities with the wider public such as science-themed theatre [12, 16, 18] and public engagement activities such as science café talks [6, 7] and PoST events.

In 2017, we reported our initial reflections on PoST 2017 events based on information such as attendance numbers, and comments on sticky notes and social media [19]. Building on this work, we set out to evaluate more systematically events conducted in 2018. This paper describes the results of the quantitative questionnaires, supplemented by interviews and focus group discussions conducted with audience members, speakers and organizers of PoST 2018.

## Materials and methods

### Pint of Science Thailand 2018 events

In 2018, we conducted PoST at four different locations in central Bangkok, Thailand held on 1^st^ February, 15^th^, 16^th^, 17^th^ and 19^th^ May and 23^rd^ August 2018. The May event coincided with the international Pint of Science 2018 festival. The February and August events were one-night only, stand-alone events which we called Pint of Science “Shots” to differentiate them from the May 2018 event. Overall, there were 18 talks held over six event-days in 2018. Talks were delivered in English on five event days, and in Thai on one event day (19^th^ May). Talks were between 12 to 20 minutes in length followed by discussions (Table 1). There were intermissions between talks so that audience members can interact with speakers, mingle and get snacks and drinks. Unlike other Pint of Science (PoS) events, all PoS events in Thailand were free in order to encourage participation from a wide range of audiences including students. We also provided a small snack and a soft drink to each attendee.

**Table 1:** Pint of Science events and the details.

Date	Location	Events description
1^st^ February, 2018	Ari, Bangkok	**How does travel impact infection?** by Dr. Ipsita Sinha from Mahidol-Oxford Tropical Medicine Research Unit, Bangkok
		**You are what you eat** by Dr. Thanik Lertcharnrit, Department of Archeology, Silpakorn University
		**Numbers** by Prof. Sir Nicholas White from Mahidol-Oxford Tropical Medicine Research Unit, Bangkok
May 15^th^, 2019	Ari, Bangkok	**Survival of *Leptospira *and *Burkholderia pseudomallei *in common drinks** by Dr. Vanaporn Wuthiekanun, Department of Microbiology, Mahidol-Oxford Tropical Medicine Research Unit, Bangkok
		**Signatures of Selection** by Prof. Olivo Miotto, Centre for Genomics and Global Health, Mahidol-Oxford Tropical Medicine Research Unit, Bangkok
		**Resistance to antibiotics: slowing down to win the race** by Dr. Thomas Althaus, Economic and Translational Research Group, Mahidol-Oxford Tropical Medicine Research Unit, Bangkok
May 16^th^, 2018	Ari, Bangkok	**Why is the sky dark at night?** by Dr. Rob Knoops from Particle Physics Research Laboratory, Chulalongkorn University
		**Bacterial morphology: why bacteria care how they look?** by Ms. Suparat Giengkam, Department of Microbiology, Mahidol-Oxford Tropical Medicine Research Unit, Bangkok
		**Controlled Human Malaria Infection studies** by Prof. Nicholas PJ Day, Director, Mahidol-Oxford Tropical Medicine Research Unit, Bangkok
May 17^th^, 2018	Ari, Bangkok	**Turning Passion in Enzymes into Innovations** by Prof. Pimchai Chaiyen, Mahidol University & Vidyasirimedhi Institute of Science and Technology
		**Invasive species, New Guinea Flatworm and Its Potential Role of Eosinophilic Meningoencephalitis in Thailand** by Dr. Kittipong Chaisiri, Department of Helminthology, Mahidol University
		**Hidden Messages behind Beautiful Biological Structures** by Dr. Puey Ounjai, Center of Nanoimaging & Department of Biology, Mahidol University
May 19^th^, 2018	Bang Rak, Bangkok	**Sea-level Changes and Its Problematics on Studying Dvaravati Culture** by Dr. Trongjai Hutangkura, Princess Maha Chakri Sirindhorn Anthropology Centre
		**HIV/AIDS: Myths vs Evidence** by Dr. Rapheephan Maude, Department of Epidemiology, Mahidol-Oxford Tropical Medicine Research Unit, Bangkok
		**What we know and what we should know about research and fieldwork** by Ms. Ravikanya Praphasavat, Mahidol-Oxford Tropical Medicine Research Unit, Bangkok
23^rd^ August, 2018	Ploenchit, Bangkok	**Pandora's Pets** by Dr. Matthew Robinson from Lao-Oxford-Mahosot Hospital Wellcome Trust Research Unit, Vientiane, Laos.
		**The sex lives of malaria parasites** by Dr. Andrea Ruecker, Mahidol-Oxford Tropical Medicine Research Unit, Bangkok.
		**How do archaeologists know where to dig?** by Dr. Noel Hidalgo Tan from Southeast Asian Regional Centre for Archaeology and Fine Arts (SEAMEO SPAFA), Bangkok, Thailand

Events were advertised on the PoST website (www.pintofscienceth.com) and social media platforms such as Facebook, Twitter and the PoST Meetup page (https://www.meetup.com/Pint-of-Science-Thailand/). Emails were also sent out to previous PoST attendees, the MORU internal mailing list and personal mailing lists of the organizing committee members and speakers.

Potential speakers were invited by the organizing committee members and through personal contacts. For the PoST 2018 main event in May 2018, talks were grouped according to the themes chosen by the PoS international team: Infection, Microwaves to Microbes, Our World and Humans [4]. Speakers were provided standard PoS guidelines on how to prepare their talks around a month before the event and were advised to prepare 10–12 minute talks with minimal number of presentation slides, and minimize/explain the use of jargons or technical terms. No formal training was provided. However, guidance on how to prepare the talks including the suggestions on the content and practice sessions were offered based on the case-by-case assessment. A member of the PoST organizing committee served as the Scientific Content Manager (AR) and as a point-of-contact and scientific content curator to all speakers.

### Quantitative data collection

#### Questionnaire design

A self-reported questionnaire was designed in English following published guidelines on designing questionnaires for evaluations [20, 21]. The questions were derived from reviews of published literature [21], mostly reports and guidance on evaluation of public engagement activities [22–24]. Apart from demographic and general questions (questions 1–8), the questionnaire consisted of questions related to motivation of attending PoST (question 9), level of participation (questions 10–12), knowledge and interest in science (questions 13–15), enjoyment (question 16) and level of satisfaction with various aspects of the event (question 17) (S1 Table). There were additional questions for speakers to provide their feedback on their experience as speakers. The draft questionnaire underwent pilot testing among PoST committee and audience members to resolve the issues of ambiguity and incomprehensibility. The final version of the questionnaire was translated to Thai. Both the final English and Thai versions were used in the evaluation. Participants were given the option of leaving their contact details should they wish to be contacted to participate in the semi-structured interviews (SSI) and a focus group discussion (FGD) for more in-depth discussions (S2 and S3 Tables).

#### Respondents of the questionnaire

The quantitative evaluation was conducted for all events in May and August 2018 (not February 2018 because the questionnaire was not ready yet at the time). Based on the registration logbook, a total of 180 entries were recorded over five event days. This number included speakers, organizing committee and audience members. All attendees at the May and August events were handed the questionnaire printed on A4 paper in either Thai or English, and a pen at registration.

#### Data management and analysis

All collected data were entered into a Microsoft Excel database and were analyzed using the IBM Statistical Package for the Social Sciences (SPSS) version 24.0, Armonk, NY: IBM Corp. The descriptive analyses included descriptive statistics such as frequency, mean and percentage. Inferential analyses were made to calculate the total median score on their overall ratings using a likert scale on following variables (questions 14–17): 1. Respondents learnt new knowledge from the events (score range = 0–5); 2. Current event increased their interest in science (score range = 0–5); 3. Enjoyed the event (score range = 0–5); and 4. Satisfaction in terms of venue (score range = 0–5); speakers (score range = 0–5); timing (score range = 0–5); content (score range = 0–5); and opportunity for interactions (score range = 0–5). The calculated overall score derived from the likert scale questions is termed as “audience score” in this article and is treated as a dependent variable. Minimum and maximum value for audience score ranges from 0 to 40. Median values of audience score were compared between various categories of participants (treated as independent variables; questions 1–13), for example age groups, gender, previous visits to other MORU-organized events and the responses to questions such as ‘yes’ and ‘no’.

Mann Whitney U and Kruskal Wallis tests were used to test the differences in median scores. In addition, one specific variable that was designed to assess respondents’ prior knowledge (question 13) and learnt knowledge on presented topic (question 14) was analysed using t-test (it was treated as a before and after variable and mean score was compared) for repeated measure. The difference in mean and median scores were statistically considered significant if p-value was less than 0.05.

### Qualitative data collection

#### Semi-structured interview (SSI) guide and focus group discussion (FGD) guide

A semi-structured interview guide (SSI) and a focus group discussion (FGD) guide consisted of the following themes (1) entertainment value of the PoST events, (2) general benefits of the event, (3) changes felt by respondents at individual level and (4) recommendations for improvements.

#### SSI and FGD participants

All participants who agreed to be contacted were invited to participate in an SSI or a FGD. Of those invited, fourteen accepted the invitation. All SSIs and FGDs were conducted at a time and location convenient for the participants (Table 2). Of the fourteen who accepted the invitation, eleven participated in SSIs and three participants (they came together) opted for a FGD. Two authors (BA and PHH) interviewed these participants in English. All interviews were audio-recorded and transcribed verbatim. All the transcripts were analysed (coded line by line) using pre-set themes described above using QSR Nvivo Qualitative software. Any emerging themes were identified and coded.

**Table 2:** Demographics of SSI and FGD participants.

ID	Gender	Age (years)	Nationality	Occupation
SSI1	Female	25–35	Thai	Science field
SSI2	Male	20–30	Thai	Science field
SSI3	Male	30–40	American	Businessman
SSI4	Female	20–30	Thai	Science field
SSI5	Male	25–35	British	Science field
SSI6	Female	25–35	Thai	Science field
SSI7	Female	45–50	Australian	Training consultant
SSI8	Female	20–30	Thai	Science field
SSI9	Male	25–35	Thai	Science field
SSI10	Female	25–30	Thai	Science field
SSI11	Female	25–35	British	Science field
FGD	2 Female, 1 Male	20–30	All Thai	Science field

### Ethical approval and consent to participate

This initiative was considered as a program/service evaluation and therefore was exempted from ethics review by Oxford Tropical Research Ethics Committee, University of Oxford. Oral informed consent was obtained from each participant before the interviews or focus group discussions were conducted.

## Results

### Quantitative methods

#### Registration

At registration, we collected the following details from each attendee: name, institution and contact number or email address. A total of 267 participants were registered for the six events. A total of 69 participants were registered in the February event, 49 on 15^th^ May, 2018, 27 on 16^th^ May, 36 on 17^th^ May, 13 on 19^th^ May and 55 on 23^rd^ August, 2018). The single-night events were most well attended and the event held in Thai language had the fewest number of attendees.

#### General characteristics of questionnaire respondents

Of the 180 questionnaires handed out, only 125 attendees completed the questionnaire. A total of 55 participants either did not complete the questionnaire or only partially completed it. An equal proportion of male and female participated in the questionnaire (male: 63/125; 50.4%, 61/125; 48.8% and other: 1/125; 0.8%) (Table 3). The mean age of the participants was 34 years and the age ranged between 20 to 78 years. Almost half (61/125; 49.6%) of the participants were Thai. The majority of participants were Asians (83/125; 67.5%), and almost half of them work or study in science-related fields (67/125; 53.6%).

**Table 3:** Total audience's scores in relation to their characteristics (n = 125).

Characteristics	Number (%)	Median score	p-value
**Gender**			
Male	63 (50.4)	34.3	0.3
Female	61 (48.8)	35.1	
Other	1 (0.8)	34	
**Age Group**			
≤31 years	70 (56)	35.4	0.06
≥32 years	55 (44)	33.8	
Mean = 34±11.56, median = 30, min = 20 and max = 78			
**Nationality**			
Thai	61 (49.6)	34.6	0.51
Non-Thai	62 (50.4)	34.8	
**Continent**			
Asian	83 (67.5)	34.4	0.18
African	3 (2.4)	37.3	
European	14 (11.4)	35.9	
North American	22 (17.9)	34.7	
Australian	1 (0.8)	35	
**Institution**			
From Thailand	77 (61.6)	34.9	0.95
Not from Thailand	8 (6.4)	35.2	
Not reported	40 (32)	34.3	
**Occupation**			
Related to Science	67 (53.6)	34.2	0.58
Unrelated to Science	58 (46.4)	35.3	
**Travel time in mins**			
≤30 mins	79 (66.4)	34.9	0.9
≥31 mins	40 (33.6)	34.6	
**Heard about the events from**			
**Friends**			
Yes	54 (43.2)	34.9	0.53
No	71 (56.8)	34.5	
**Facebook**			
Yes	34 (27.2)	34.5	0.76
No	91 (72.8)	34.8	
**Twitter**			
Yes	1 (0.8)	34.7	0.91
No	124 (99.2)	40	
**Meetup**			
Yes	15 (12)	33.8	0.9
No	110 (88)	34.8	
**Organizers**			
Yes	24 (19.2)	35.6	0.42
No	101 (80.8)	34.5	
**Website**			
Yes	4 (3.2)	34.7	0.76
No	121 (96.8)	34.7	

Median score = 35, Min = 18 and Max = 40

#### Knowledge about the event and previous attendance

With regard to how attendees have heard about the events, the most frequent source of information was from friends (54/125; 43.2%), followed by Facebook (34/125; 27.2%), PoST organizing committee members (24/125; 19.2%) and the PoST Meetup website (15/125; 12%) (Table 3). Of the 125 participants, a quarter (32/125; 25.6%) had attended the PoST 2017 events, followed by PoST Shots event in February 2018 (13/125; 10.4%), Science Cafés (7/125; 5.6%) and the MORU-organized Thai Geographic Information System meetups (https://thaigis.net/) (6/125; 4.8%).

#### Reasons for the participation and perceived benefits of attending Pint of Science events

**Fig 1:**
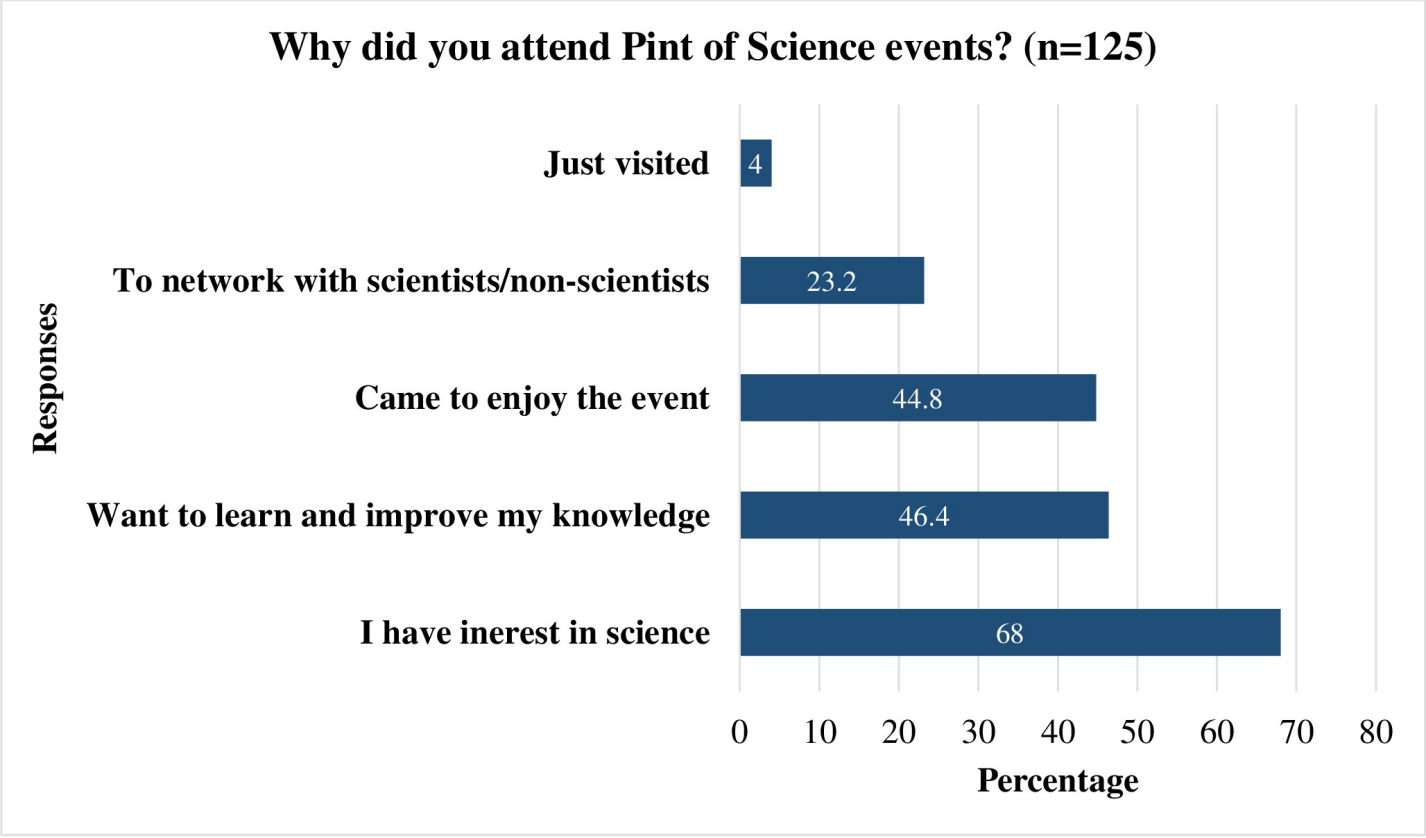
Responses by attendees about the reasons for participation. The figure consists of ‘Yes’ responses to the options presented.

When asked about the reasons for participation, the following were the most frequent responses: “Interested in science” (85/125; 68.0%), “want to learn and improve my knowledge” (58/125; 46.4%), and “came to enjoy the event” (56/125; 44.8%) (Table 4 and Fig 1). Majority of attendees responded that they made new contacts (87/125; 69.6%) and exchanged scientific knowledge (73/125; 58.4%). Respondents were also able to network with both scientists and non-scientists (29/125; 23.2%) and many participated by asking questions (35/125; 28.2%).

**Table 4:** Total audience's scores in relation to their experience and feedback (n = 125).

Characteristics	Number (%)	Median score	p-value
**Have attended following events in past**			
**PoST 2017**			
Yes	32 (25.6)	34.5	0.73
No	93 (74.4)	34.8	
**PoST Shots (February 2018)**			
Yes	13 (10.4)	34.8	0.84
No	112 (89.6)	34.7	
**Science Cafes**			
Yes	7 (5.6)	36.1	0.77
No	118 (94.4)	34.6	
**Thai GIS**			
Yes	6 (4.8)	32.5	0.31
No	119 (95.2)	34.8	
**Did not attend any**			
Yes	80 (64)	34.8	0.31
No	45 (36)	34.5	
**Why do you attend?**			
**I have interest in science**			
Yes	85 (68)	35.2	**0.03**
No	40 (32)	33.6	
**Came to enjoy the event**			
Yes	56 (44.8)	35.5	**0.02**
No	69 (55.2)	34	
**Want to learn and improve my knowledge**			
Yes	58 (46.4)	35.5	**0.01**
No	67 (53.6)	34.0	
**To network with scientists/non-scientists**			
Yes	29 (23.2)	35.7	0.28
No	96 (76.8)	34.4	
**Just visited**			
Yes	5 (4)	32.4	0.49
No	120 (96)	34.8	
**Did you make new contacts?**			
Yes	87 (69.6)	35.1	0.27
No	36 (28.8)	34	
Other	2 (1.6)	29	
**Did you exchange scientific knowledge?**			
Yes	73 (58.4)	35.5	0.22
No	51 (40.8)	33.7	
Other	1 (0.8)	27	
**Did you ask questions?**			
Yes	35 (28.2)	34.6	0.53
No	88 (71)	34.8	
Other	1 (0.8)	27	

Median score = 35, Min = 18 and Max = 40

#### Overall audience scores and associated factors

**Fig 2:**
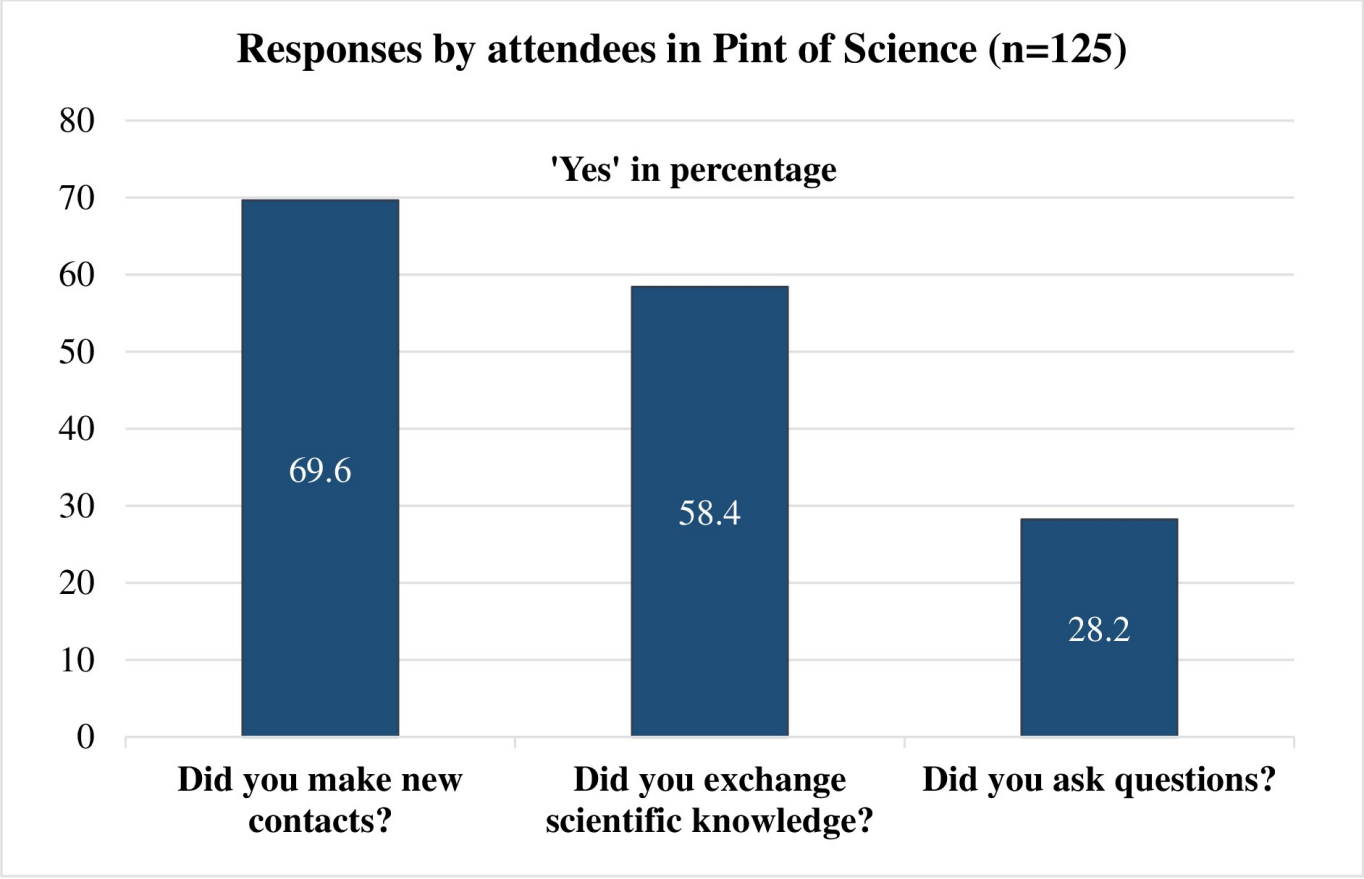
Responses to questions by attendees. The figure consists of ‘Yes’ responses to the questions asked.

Respondents were asked to rate the event on a likert scale (each question’s score ranged from 0 to 5) based on the following: if they learnt any new knowledge from the event, the event enhanced their interest in science, the extent they enjoyed the event and overall ratings on the venue, speakers, timing, content and opportunity for interactions (Table 4 and Fig 2). The total maximum score was 40, minimum was 18, and the median score was 35. The reasons for participation which showed higher median scores were: 1. “I have an interest in science” (Yes: median score 35.2 versus No: 33.6; p-value = 0.03), 2. “Came to enjoy the event” (Yes: median score 35.5 versus No: 34.0; p-value = 0.02) and 3. “Want to learn and improve my knowledge” (Yes: median score 35.5 versus No: 34.0; p-value = 0.01).

#### Knowledge before and after the event

**Fig 3:**
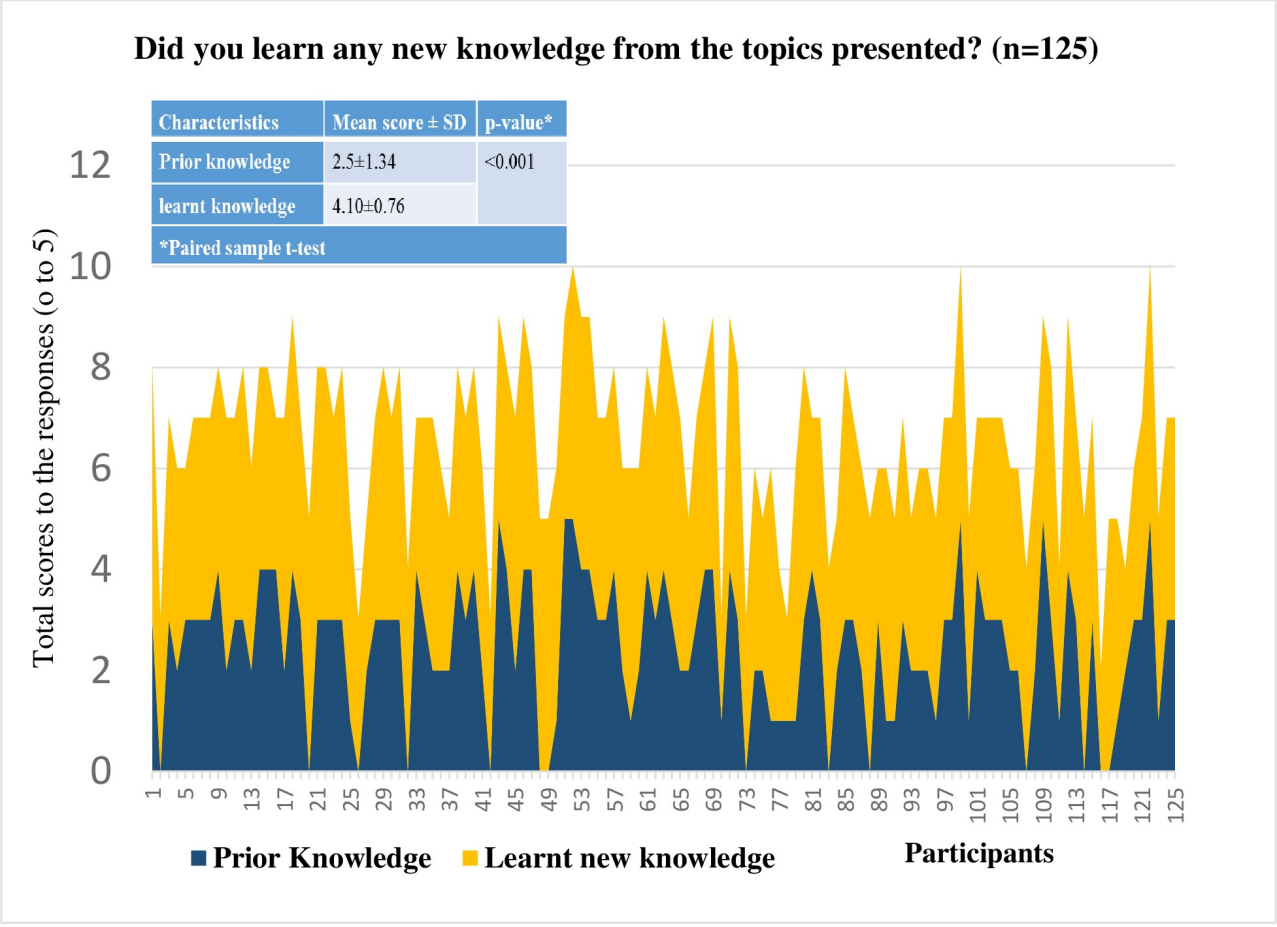
Prior knowledge and learnt knowledge about the topics presented in Pint of Science events.

In response to whether attendees had previous knowledge on the topics presented and if they learnt new knowledge related to the topic, a significant difference in mean score was noted (prior knowledge: mean score = 2.5 versus learnt new knowledge: mean score = 4.1; p-value<0.001) (Fig 3).

#### Qualitative methods

A total of 11 attendees (7 women and 4 men) participated in semi-structured interviews (SSIs) and 3 attendees participated in a FGD (Table 2). The results of SSIs and FGDs as well as the free text section of the questionnaire are presented according to three themes: entertainment value, benefits of the event, and recommendations for future improvements.

#### Entertainment value

Audience members appreciated the entertainment value of the event. The majority of people we interviewed enjoyed the event. Their experiences were often related to the way the presentations were made in terms of how speakers chose simple language together with the relaxing atmosphere i.e. non-formal settings with snacks and soft drinks provided.

*I think so far*, *[including] last year and this year and yesterday speakers [all] three of them, I think they are pretty entertaining so I think they are the main sources of the entertainment for this event really. That makes really well. They're entertaining by them self and I think you should choose right people not only for their knowledge but also for their entertainment. That's really nice and I think you also do a good job for not only entertainment but relaxing people. I think people [were] calm, easy….. and the environment and simple snacks that [were] provided so [that] people don't have to hang out on an empty stomach that's really relaxing. I think the combination is good for entertaining.*

25–35 year old, female, engineer, audience member, SSI

Audience members also commented on how science lectures ought to be blended with arts, entertainment and story-telling in order to convey the message and make science more interesting.

*…………..yeah that's your part of resume, when you went to the school, undergrad or graduate program they don't give you like an entertainment value but for science to get a bigger audience the scientists should be a better entertainer, better storyteller to help carry the rest of the people that's how people work*.

35–40 year old, male, businessman, audience member, SSI

#### Benefits of the event

Several benefits were mentioned by audience members for example how speakers were able to help them understand complex scientific concepts. In addition, audience members appreciated how science was explained in a way that is applicable in their day-to-day lives. For instance, the evolutionary adaptation of bacteria and its relevance to understanding of superbugs was found to be interesting.

*I do love the way the speakers picked interesting topics, mostly hard to understand by reading it myself, and clarify it in the easiest way. It can make people love science more, as science can explain many facts in our daily living. For example, why this bacteria have to transform their structure for living*.

25–30 year old, male, science student, audience member, SSI

Consider this quote by an audience member who was referring to a talk on antimicrobial resistance. She explained how beneficial it was to be aware of the harms of misusing antibiotics.

*The lay person didn’t know much about the antibiotics, but from this event*, *they will know how and when they should use antibiotics*

20–25 year old, female, non-science student, audience member, FGD

Many speakers said that they benefited from participating in PoST by improving their communication skills and having the opportunity to network with scientists and non-scientists. In addition, speakers mentioned that such events were a good means to engage with the public, can improve the visibility of their work and potentially attract more funding.

*You can look at the benefits for the speakers; it increases their profile. And you could say you contributed in some sort of public engagement……..You know it might sound selfishly but if people know your research more, you probably have chance to receive more funding*,

30–35 year old, female, research physician, presenter, SSI

In addition, audience members mentioned how such events were helpful in providing information on topics that they would not normally be exposed to.

*I have to say to be fair it was an opportunity for me to be exposed to other topics I wouldn’t necessarily go out of my way to look and try to discover and understand more. So these events have… sort of many benefits…. I guess, you cannot express everything… but you are also a little bit of awestruck while other people talking to you, teaching you the new things you didn’t know before. That’s why I found out very good and interesting*.

25–30 year, male, physician, audience member, SSI

Some members of the audience members also highlighted that “live” events have benefits that cannot be found from other sources such as the internet. They particularly enjoyed the interactive nature of the talks.

#### Recommendations for future improvements

Many audience members were happy about the organization of the event in terms of speakers, venue and the benefits of such events. Nevertheless, on prompting how we could improve such events in future, audience members had the following suggestions.

*I think the topics are very (very) great and those are interesting but make sure to include more variety in topic range because what I have listened on that event is mostly about medical science and from MORU so I think maybe we have to include more stuffs like different topics from different Institute and we can make it more varied in range*.

20–25, male, science student, audience member, FGD

In addition, many audience members suggested that we should increase our promotion of the events among university students and on social media.

*One thing I have to suggest to make it better for this event is that to make more people know this event maybe you should get a contact with some institutes in your area like Chulalongkorn University or any other. You know that this is the good thing or maybe you're running this event with one of the speakers who have given the talk and you post that talk on Facebook. Or maybe you can make some people watch [in Facebook/Youtube] and then they feel like all these events are cool and they will be like I would like to get there to receive some interesting topics tomorrow or maybe other day*.

20–25, male, science student, audience member, FGD

Other recommendations were related to some of the technical and logistics aspects such as improvements on audio-visuals (e.g. microphone) and chair arrangements (need for more spacious arrangement). The common recommendations for all events were more availability of (free) beverages, need for bigger space, longer breaks in between talks and accessibility by public transport such as the Bangkok Sky Train (BTS).

## Discussion

Globally, despite the growing number of public engagement activities, there is still a lack of published literature on the evaluation of such events. In 2018, Pint of Science (PoS) was held in 21 countries but as far as we know, this is the first paper that reports the quantitative and qualitative evaluation results from both organizers and the audience members who participated. Previous PoS publications consisted mainly of reflections from the organizing committee [2, 19].

The event held in Thai had the fewest number of attendees despite that they were advertised in both Thai and English on Pint of Science web pages and held in central Bangkok [4]. One explanation was that most scientific work and literature is in English, and that most people interested in science speak and work professionally in English. In 2017, we intended to hold the PoST events in dual language with at least one talk per night in Thai with hosting done in both Thai and English [4]. However, because all audience members could understand English, two out of three Thai language presenters decided to present their talks in English despite having prepared their talks in Thai.

Another possible reason for low attendance for the Thai language event is the time and location. It was held on a Saturday afternoon and a different part of the city from the English language events. Location and environment have been identified as determinants for high or low attendance figures [8]. For example, “Nerd Nite” events in Hong Kong have been very well attended due to the location of the events—a bar right in the heart of the business and finance district [8].

Although assessments were not specifically made in regards to socio-economic status of the participants, in general the PoST events were participated by those who were affiliated to science related fields or the participants who speak and understand English. This suggests that such events primarily attract audiences from middle to higher income group, and have higher level of education. This phenomenon has been highlighted by others [25].

Both the survey and qualitative component of the evaluation showed that audience members attended PoST because of their interest in science, its entertainment value and their desire to learn; our study shows that audience members indeed enjoyed the event and perceived that they have increased their knowledge. These findings were consistent with evaluations of similar initiatives as the science café-like programs in Cambodia [6], Hong Kong [8], USA [26, 27], the Netherlands [28] and participation in science festivals in the UK [29, 30].

Both attendees and presenters expressed that PoST events provided an opportunity for interaction between scientists and the public. The many questions from the audience and the lively discussions following the talks reinforces this finding. This is consistent with the aims of PoS which is to “provide a platform which allows people to discuss research with the people who carry it out and no prior knowledge of the subject is required” [1].

For presenters, the experience of presenting their work to a lay audience had the benefit in that they could improve their presentation and communication skills, expand their network, enhance their public profile, and potentially increase their opportunities for future funding. These benefits have been frequently echoed around the globe in relation to public engagement activities [31–33]. Others have noted that for junior researchers, having public engagement experience is an advantage in their CV [2].

This study also showed that audience members perceived that they gained knowledge on specific topics presented. The perceived gain in knowledge does not necessarily mean that they actually gain knowledge or translate their knowledge to behavior change, however, the perceived change in itself is highly valued aspects of such events [29, 34]. It is also likely that the perceived knowledge gain or the changes may be minimal to an audience member who has high prior knowledge and may not learn new knowledge from the topics presented. In addition, the gain in knowledge in the topics presented does not necessarily reflect the critical scientific literacy amongst audience [35]. Although public engagement events such as PoST contribute to science literacy, in general such events may not equip audience with critical scientific literacy necessary to understand the mechanisms how science related evidence are produced, their interpretations and the caveats [35].

Critical evaluation through recommendations and feedback of the PoST events by participants is indispensable for future improvements. Many of those we interviewed suggested that one way to increase the reach and range of topics and its diversity is to increase collaborations with local research institutions and universities. These findings are consistent with recommendations from the PoST team [2].

Other suggestions were related to the practicalities and logistics of holding the events, for example many wanted bigger venues that are easily accessible by public transport (BTS in the case of Bangkok), and longer break-time for interactions between scientists and audience members. Events with longer break time with venues accessible to the general population were found to be conducive factors to enhance the participation in Hong Kong [8]. These suggestions will be incorporated into the planning of subsequent years’ events.

This study has several strengths. As far as we know, this is the first formal evaluation of PoST. Findings were triangulated by using both quantitative and qualitative methods. In order to minimize social desirability bias, the questionnaires were self-administered. In addition, all SSIs and FGDs were conducted in locations chosen by the interviewee and often in non-formal settings such as cafés.

This study also has several limitations. Since the data were derived from those who participated in PoST activities, self-selection bias was inevitable. In addition, this was a cross-sectional study and therefore, the data represent the immediate impression about these activities. We also acknowledge that the evaluation was conducted by the organizers of PoST, and therefore not independent. The findings from this study need to be interpreted cautiously as the feedback and reflections presented in this study may be applicable to these self-selected groups of attendees. In future, we may consider having independent evaluators.

## Conclusion

Pint of Science Thailand was found to be enjoyable by speakers and audience members alike. Audience members found the events to be beneficial in that they gained additional knowledge, got the opportunity to interact with scientists and discuss science. To improve PoST activities, audience members suggested to include a diverse range of topics, more collaborations with other local research institutions and bigger venues. PoST was well received with recommendations to improve minor issues related to practicalities and logistics and can be applicable for various other public engagement events conducted worldwide. This study reinforces the benefits of public engagement events in increasing interest in science, building literacy around it, bridging the gaps between scientists and the public, and building capacity of scientists in communication and translation of their work.

## Supporting information

S1 Table Pint of Science Thailand questionnaire. (PDF)

S2 Table Pint of Science Thailand SSI guide. (DOCX)

S3 Table Pint of Science Thailand FGD guide. (DOCX)
