# Supplementary material for: Evaluation of the Pint of Science festival in Thailand
Source: PLoS One. 2019 Jul 18;14(7):e0219983. doi: 10.1371/journal.pone.0219983 (PMC6638989; doi:10.1371/journal.pone.0219983)
Supplement: S1 Table — (PDF) [file pone.0219983.s001.pdf]

We would be delighted to get your feedback on the event today. Please complete the form as appropriate.

1. **Gender:** ○ Male ○ Female ○ Other
3. **Nationality:** ………………………………….
2. **Age**: ………….. (years)
4. **Institution:** ………………………………….

5. **What is your primary occupation?**

○ Science student ○ Non-science student ○ Scientist ○ Non-scientist ○ Retiree

○ Other (*Specify*…………….…………….………………….)

6. **How long did you travel today to reach to this venue?** ……………………….(approximate time in minutes)

7. **How did you hear about this event?**

○ Friends ○ Facebook ○ Twitter ○ Eventbrite ○ Meetup ○ Organizers

○ Website (*Specify*………….……………….…….………….) ○ Other (*Specify*……………………........….….)

8. **Have you attended any of these science events?**

☐ Pint of Science Thailand 2017 ☐ Pint of Science (in other country) ☐ Pint of Science Shots

☐ Science café ☐ Thai GIS ☐ Other (*Specify*…………….………….….…) ☐ None of the above

9. **Why did you attend this event?**

☐ I am interested in science topics ☐ I want to meet and network with scientists/researchers

☐ I came to enjoy the event ☐ I just visited this venue and found there was an event today

☐ I want to learn and improve my knowledge ☐ Other (*Specify*………………………..……………..….)

10. **Did you make new contacts?**

○ Yes ○ No ○ Other (*Specify*………..……….……………………………………………………….)

11. **Did you exchange scientific knowledge?**

○ Yes ○ No ○ Other (*Specify*…………………………...…………..…….….…………….….)

12. **Did you ask any questions related to the topic presented to the speakers?**

○ Yes ○ No ○ Other (*Specify*…………………………..………………………………..……………..….)

Using a scale of 0= none and 5=high; please rate the impact of this event on the following categories:

13. **What level of prior knowledge did you have on the event's topics?** 0 1 2 3 4 5

14. **Did you learn any new knowledge from the talks?** 0 1 2 3 4 5

15. **Did this event increase your interest in science?**	0	1	2	3	4	5
16. **Did you enjoy the event?**	0	1	2	3	4	5
17. **Are you satisfied with this event?**						
17.1 Venue	0	1	2	3	4	5
17.2 Speakers	0	1	2	3	4	5
17.3 Timing	0	1	2	3	4	5
17.4 Content	0	1	2	3	4	5
17.5 Opportunity for interactions	0	1	2	3	4	5
18. ***For Speakers only***						
18.1 Improved skills in preparing and delivering talks	0	1	2	3	4	5
18.2 Inspiration to continue working in science	0	1	2	3	4	5
18.3 Inspiration to continue with public engagement	0	1	2	3	4	5

A. In your opinion, can you tell us what worked well and what did not in terms of these events?

B. Please provide any suggestions for how this event could be improved?

Thank you very much!

Please visit us online at: https://www.pintofscienceth.com/; Facebook: https://www.facebook.com/pintofscienceTH/
Phone (office): +66 22036333; mobile: +66 (0) 619864025
