# Supplementary material for: Evaluation of the Pint of Science festival in Thailand
Source: PLoS One. 2019 Jul 18;14(7):e0219983. doi: 10.1371/journal.pone.0219983 (PMC6638989; doi:10.1371/journal.pone.0219983)
Supplement: S2 Table — (DOCX) [file pone.0219983.s002.docx]

Themes

(SSIs will cover the individual perspective on public engagement events that will cover the themes such as entertainment, knowledge, attitude/practice and recommendation).

Part I: Specifics of the event

| Note takers’ initials |  | | | | | | |
| --- | --- | --- | --- | --- | --- | --- | --- |
| Date and time |  | | | | | | |
| Location |  | | | | | | |
| Name of the event |  | | | | | | |
| Name/Code | Age | Sex | Education | Occupation | City/Country | Event attended | Remarks |
|  |  |  |  |  |  |  |  |

Part II: Themes/Values of PE

| Theme I: Entertainment |
| --- |
| Can you tell us your experience about the event (please introduce/remind them the event you are talking about)?  Can you tell us whether you enjoyed the event or not? (if “yes” and “no”, can you give us the reasons?) |
| Theme II: Benefits of the event? |
| Did you feel comfortable attending the event?  Do you think this event was helpful in disseminating science, research or any new knowledge? (Please can you provide us reasons for “yes” and “no”?) |
| Theme III: Changes in you and overall changes such events bring |
| Do you think such events are useful? In what way are they useful?  Would you participate again in such events in future? If so why?  Would you recommend your friends or relatives to attend these events in future? If so, why? |
| Theme IV: Recommendations/Future improvements (Please also allow them to free talk on what they thought to improve for future) |
| Do you have any recommendations for us to improve such events? If so what are they? |

Thank you!
