# Supplementary material for: Evaluation of the Pint of Science festival in Thailand
Source: PLoS One. 2019 Jul 18;14(7):e0219983. doi: 10.1371/journal.pone.0219983 (PMC6638989; doi:10.1371/journal.pone.0219983)
Supplement: S3 Table — (DOCX) [file pone.0219983.s003.docx]

Themes

(PE events generally have following values which will be covered by FGDs).

Part I: Specifics of the event

| Note takers’ initials |  | | | | | | |
| --- | --- | --- | --- | --- | --- | --- | --- |
| Date and time |  | | | | | | |
| Location |  | | | | | | |
| Name of the event |  | | | | | | |
| Number of participants |  | | | | | | |
| Name/Code | Age | Sex | Education | Occupation | Address | Event attended | Remarks |
|  |  |  |  |  |  |  |  |
|  |  |  |  |  |  |  |  |
|  |  |  |  |  |  |  |  |
|  |  |  |  |  |  |  |  |
|  |  |  |  |  |  |  |  |
|  |  |  |  |  |  |  |  |
|  |  |  |  |  |  |  |  |
|  |  |  |  |  |  |  |  |
|  |  |  |  |  |  |  |  |
|  |  |  |  |  |  |  |  |

Part II: Themes/Values of PE

| Theme: Entertainment (please ask if these events were entertaining to them) |
| --- |
| Can you tell us your experience about the event (please introduce/remind them the event you are talking about)?  Can you tell us whether you enjoyed the event or not? (if “yes” and “no”, can you give us the reasons?) |
| Theme II: Please ask if such events added any new knowledge or contributed in familiarizing the science, disseminating it and creating the platform for discussion |
| Did you feel comfortable attending the event?  Do you think this event was helpful in disseminating science, research or any new knowledge? (Please can you provide us reasons for “yes” and “no”?) |
| Theme III: Please ask if such events had an impact on them, for example their attitude/practice |
| Do you think such events are useful? In what way are they useful?  Would you participate again in such events in future? If so why?  Would you recommend your friends or relatives to attend these events in future? If so, why? |
| Theme IV: Recommendations/Future improvements (Please also allow them to free talk on what they thought to improve for future) |
| Do you have any recommendations for us to improve such events? If so what are they? |

Thank you!
